# Bcl9 Depletion Modulates Endothelial Cell in Tumor Immune Microenvironment in Colorectal Cancer Tumor

**DOI:** 10.3389/fonc.2020.603702

**Published:** 2021-01-19

**Authors:** Zhuang Wei, Mei Feng, Zhongen Wu, Shuru Shen, Di Zhu

**Affiliations:** ^1^ Department of Pharmacology, School of Pharmacy, Fudan University, Shanghai, China; ^2^ Key Laboratory of Systems Biology, Innovation Center for Cell Signaling Network, CAS Center for Excellence in Molecular Cell Science, Institute of Biochemistry and Cell Biology, Shanghai Institutes for Biological Sciences, Chinese Academy of Sciences, Shanghai, China; ^3^ Department of Pharmacology, Shanghai Pudong Hospital, Fudan University Pudong Medical Center, Shanghai, China; ^4^ Key Laboratory of Smart Drug Delivery, Ministry of Education, & State Key Laboratory of Molecular Engineering of Polymers, School of Pharmacy, Fudan University, Shanghai, China; ^5^ Shanghai Engineering Research Center of ImmunoTherapeutics, Fudan University, Shanghai, China; ^6^ Yangtze Delta Drug Advanced Research Institute, Nantong, China

**Keywords:** tumor immune microenvironment, colorectal cancer, endothelial cell, BCL9, Wnt pathway

## Abstract

Tumor endothelial cells are an important part of the tumor microenvironment, and angiogenesis inhibitory therapy has shown potential in tumor treatment. However, which subtypes of tumor endothelial cells are distributed in tumors, what are the differences between tumor endothelial cells and normal endothelial cells, and what is the mechanism of angiogenesis inhibitory therapy at the histological level, are all need to be resolved urgently. Using single-cell mRNA sequencing, we analyzed 12 CT26 colon cancer samples from mice, and found that knockdown of the downstream factor BCL9 in the Wnt signaling pathway or inhibitor-mediated functional inhibition can modulate tumor endothelial cells at a relatively primitive stage, inhibiting their differentiation into further extracellular matrix construction and angiogenesis functions. Furthermore, we propose a BCL9-endo-Score based on the differential expression of cells related to different states of BCL9 functions. Using published data sets with normal endothelial cells, we found that this score can characterize endothelial cells at different stages of differentiation. Finally, in the The Cancer Genome Atlas (TCGA) pan-cancer database, we found that BCL9-endo-Score can well predict the prognosis of diseases including colon cancer, kidney cancer and breast cancer, and identified the markers of these tumor subtypes, provide a basis for the prognosis prediction of patients with such types of tumor. Our data also contributed knowledge for tumor precision treatment with angiogenesis inhibitory therapy by targeting the Wnt signaling pathway.

## Introduction

Endothelial cells and fibroblasts are a type of tumor-related cells that are widespread in tumors. More and more experimental evidence shows that tumor fibroblasts and endothelial cells play a role in promoting the occurrence and development of tumors (1, 2). But for a long time, how the endothelial cells and fibroblasts of tumors play a role in the occurrence and development of tumors has been stuck in the metaphysical imagination, lacking a comprehensive analysis to distinguish between connotation and extension. As we all know, endothelial cells and fibroblasts are a type of cells with the same origin and the potential to differentiate. Fibroblasts are considered as progenitor cells of endothelial cells (3), but the functions of endothelial cells are obviously different from fibroblasts. And endothelial cells also have different groups and stages of differentiation inside. Therefore, which types of cells in the endothelial cell and fibroblast population play an important role in tumors, and what are the similarities and differences in the roles of these cells in different types of tumors have become an urgent question to be answered.

The Wnt gene is synonymous with Wingless gene in Drosophila and Int gene in mice, identified for its proto-oncogene function in breast tumors firstly in 1982 (4). The Wnt ligands family includes 19 secreted cysteine-rich glycoproteins and participate in many biological processes of cell fate determination, such as cell division, proliferation, and migration (5). The Wnt pathway is a highly conserved pathway that plays an important role in embryonic development and tissue homeostasis, and involved in the development of many disease, including malignant tumor (4, 6).

The Wnt signaling pathway is historically divided into canonical and noncanonical pathways. The canonical pathway is β-catenin-dependent pathway, the noncanonical pathway including Wnt/Ca2+ pathway and Wnt/polarity pathway (7). β-catenin is the most critical and core signal transduction factor in canonical Wnt signaling pathway, and is distributed in both cytoplasm and cell membrane (8). The Wnt/β-catenin signaling pathway is a process with multiple steps that include multiple proteins relocalization, phosphorylation, and degradation, further influences the transcription of target genes (8). Briefly, Without Wnt signaling extracellular, β-catenin in the cytoplasm is phosphorylated by a “destruction complex” composed of axis inhibitor (Axin), glycogen synthase kinase-3 β (GSK3β), casein kinase 1 (CK1), adenomatous polyposis coli (APC), and protein phosphatase 2 A (8). Therefore, β-catenin is recognized and ubiquitinated by E3 ubiquitin ligase β-Trcp, which binds β-catenin for proteosomal degradation (8). On the contrary, when the Wnt signaling is activated, Wnt ligands bind to Frizzled (Fzd) and LRP receptor complexes. LRP receptors are then phosphorylated by CK1α and GSK3β, thereby the Dishevelled (Dvl) proteins is recruited to the plasma membrane, which disturbs the stability of “destruction complex” and prevents the phosphorylation degradation of β-catenin (8, 9). This progress lead to the stabilization and accumulation of β-catenin in the cytoplasm, which translocate into the nucleus and contracts with LEF/TCF (lymphoid enhancer factor/T-cell factor). Moreover, β-catenin recruits co-activators (such as CBP/p300, BRG1, BCL9, and Pygo) and forms a “activation complex”, which leads to the transcription of target genes (such as CD44, VEGF, c-Myc andcyclinD1 et al.) (8, 10). This progress is tightly correlated with embryogenesis and oncogenesis.

Nuclear β-catenin, which plays a key role in the Wnt pathway, acts as a transcription factor for genes that regulate cell proliferation, migration, and survival (8). Nuclear β-catenin binds to B-cell lymphoma 9 (BCL9) and to its homologue B-cell lymphoma 9-like (BCL9L) (8, 11). BCL9 is a co-activator of the Wnt signaling pathway and regulates the recruitment of Pygopus to the nuclear β-catenin-TCF complex (8, 11). BCL9 enhances β-catenin-mediated transcriptional activity regardless of the mutational status of Wnt signaling components (12). It also promotes cell proliferation, invasion, and migration (12). BCL9 expression is very low in the normal cellular tissues from which tumors originate (12). High expression of BCL9 is often observed in many malignant tumors, including colorectal cancer, liver cancer, and breast cancer and it contributes to tumor progression, recurrence, and metastasis (12–14).

It is estimated that up to 92% CRC patients have at least one mutation in Wnt regulators reported by The Cancer Genome Atlas (TCGA) consortium in 2012 (15). Over 94% of CRC cases process at least one known protein mutation of Wnt/β-catenin pathway (15). In the majority of CRC cases, the Wnt signaling pathway mutations occur in APC gene, which is the main pathogenesis of familial adenomatous polyposis (FAP) syndrome (16, 17). Upon APC is deficient or dysregulated, the β-catenin “destruction complex” fails to be established. β-catenin accumulates and translocates to the nucleus, leading to the transcription of target genes related to CRC development (18). The APC function restoration can in turn recover crypt homeostasis and normal Wnt signaling levels in CRC murine models, regardless of the mutation of Tp53 and KRAS (19). Additionally, it is reported that about 1% of CRC cases display activating mutations of β-catenin (20, 21). The overexpression of β-catenin in the nucleus contribute to a poor outcome in CRC patients (22).

Remarkably, nearly all Wnt/β-catenin pathway mutations in CRC cause β-catenin accumulation in the nucleus eventually. Thus, targeting β-catenin/TCF interactions or inhibitors of transcriptional co-activators provide potential treatment options. B cell lymphoma 9 and its homolog B cell lymphoma 9–like (BCL9/9l) is transcriptional coactivator of β-catenin, forming part of Wnt signal enhanceosome (23). It is reported that peptides targeting BCL9/9l prevent tumor development and inhibit Wnt/β-catenin pathway activity in multiple CRC models (24). Moreover, loss of BCL9/9l suppresses CRC development driven by Wnt pathway effectively in murine models that resembling human cancer (25).

Tumor endothelial cells (TECs) are one cluster of components in tumor microenvironment. Specific tumor endothelial markers and abnormal cytogenetic expression indicate that TECs differ significantly from normal endothelial cells (NECs) (26). TECs play an important role in tumor progression and metastasis, showing both angiogenic and non-angiogenic function. Tumor angiogenesis is caused by angiogenic factors, such as vascular endothelial growth factor and basic fibroblast growth factor, which are released by tumor cells and stimulate resting endothelial cells (EC) to migrate, proliferate, differentiate, and finally form new blood vessels. Tumor blood vessels help to supply nutrients and O2 to the tumor, and meanwhile, withdraw the waste and CO2. Different from normal blood vessels, tumor vessels are unstable and loosely attached (27), which makes tumor cells easier to permeate into vessels and metastasize. In addition to forming blood vessels, TECs show a non-angiogenic function. TECs are capable to secret angiocrine factors, such as VEGF, bFGF, IL-6, IL-8 and so on, to promote tumor progression (28). TECs can also express some specific molecules, such as FasL (29), PD-L1 (30) and so on, to inhibit immune function. A study revealed the distinction between HM-TEC from highly metastatic and LM-TEC from low metastatic tumors (31) which suggests that TECs themselves take a part in tumor metastasis. Besides, circulating TECs are found to protect tumor cells from anoikis in circulation (32).

A study demonstrated that BCL9 knockdown reduced angiogenesis through down-regulation of vascular endothelial growth factor expressed by tumor cells (12). However, the potential effect of BCL9 on TECs is still unclear.

The role of extracellular substations (ECMs) in tumor micro-environments is not limited to resistance to tumor invasion. An ECM is a repository of cell binding proteins and growth factors that affect tumor cell behavior (33). It is also modified by proteases produced by tumor cells and substation cells (33). In ECMs, Wnt proteins can undergo self-secretion and side secretion of signaling proteins: Wnt ligands form gradients and act as morphological signs to determine the spatial homogeneity of target cells and affect their behavior, such as gene expression, in a concentration-dependent manner (34). Extracellular hardness and Wnt/beta-catenin signal transdivation in physiology and disease transdivine Wnt/beta-catenin signal transdivation paths play a fundamental role in development, stem cell differentiation, and steady state in the body (35), and abnormal activation may lead to disease.

In order to answer these questions, we use RNAi genetic deprivation or small molecule inhibitors to influence the BCL9 and the Wnt signaling pathway in the CT26 colon cancer mice model. Through single-cell mRNA sequencing, cluster analysis, re-clustering analysis, differential expression analysis, and biological process enrichment analysis, we found that one certain type of endothelial cells and fibroblast populations in mouse colon cancer samples were perturbed by the loss of BCL9 function. The perturbed endothelial cells tend to lose normal endothelial cell functions, and exhibit high proliferation and high cell metabolism. To quantify this impact, by using of mathematical methods, we proposed a BCL9-endo-Score endothelial cell function score based on the differential expression gene set obtained from affected and unaffected cells. We verified BCL9-endo-Score score in published endothelial cell related databases, and found this score can distinguish different stages of Endothelial cell differentiation. Finally, in the TCGA database, at the pan-cancer scale, we found that BCL9-endo-Score can predict the prognosis of one type of special marker tumors well. Our data provides a supplement to the function of endothelial cells and fibroblasts in tumors, and provides a basis for further treatment.

## Materials and Methods

### Chemicals and shRNAs

hsBCL9CT-24 was produced by AnaSpec, CA, according to previous protocols. Synthesis and purification of peptides were evaluated by analytical high-performance liquid chromatography (HPLC) and mass spectrometry (MS). hsBCL9CT-24 was dissolved as a 10 mmol/L solution; both were diluted prior to assay. pGIPZ- and/or pTRIPZ (inducible with doxycycline)-based lentiviral shRNAs for human BCL9 shRNA#3 (V3LHS_351822), mouse BCL9 shRNA#5 (V3LMM_429161), human BCL9L shRNA#4 (V2LHS_268755), mouse BCL9L shRNA#1 (V2LMM_69221), human CTNNB1 shRNA#2 (V2LHS_151023), mouse CTNNB1 shRNA#2 (V2LMM_1090), and non-targeting shRNA were obtained from Open Biosystems/GE Dharmacon. The non-targeting (NT) lentiviral shRNA construct expresses an shRNA sequence with no substantial homology to any mammalian transcript, providing a negative control.

### Tumor Specimens

Six to 8-week-old BALB/c female mice were purchased from Charles River. For BCL9-shRNA and NT-shRNA group, CT26 BCL9-shRNA and NT-shRNA cancer cells were respectively subcutaneously (s.c.) inoculated (4×10^5^ cells in PBS) in the right flank region of three mice. For Vehicle and hsBCL9_CT_-24 group, CT26 wild type cancer cells were s.c. as above in six mice. Vehicle and hsBCL9CT-24 (25 mg/kg) were intraperitoneal (i.p.) injected once every two days, respectively in three mice. For each tumor, at least four regions were sampled. Totally, 12 samples from 12 mice were collected. details information was summarized (**Table 1**). All the procedures were performed according to protocols approved by the University’s animal care committee, along with the guidelines of The Association for Assessment and Accreditation of Laboratory Animal Care International.

**Table 1:** Sample information.

ID	Age	Sex	Location	Treatment	Size(mm)	Cell Number
5	10w	Female	s.c.	Vehicle	13*10*5	6,324
8	10w	Female	s.c	Vehicle	12.2*9*4.5	7,889
10	10w	Female	s.c	Vehicle	13.4*8.6*4.3	5,073
4	10w	Female	s.c	hsBCL9_CT_-24	14.3*4.4*2.2	5,873
12	10w	Female	s.c	hsBCL9_CT_-24	11.1*4.6*2.3	6,491
16	10w	Female	s.c	hsBCL9_CT_-24	13.03*6.4*3.2	7,444
18	10w	Female	s.c	NT-shRNA	16.5*8.4*4.2	13,602
19	10w	Female	s.c	NT-shRNA	12.3*10*5	13,873
22	10w	Female	s.c	NT-shRNA	11*10*5	11,470
24	10w	Female	s.c	Bcl9-shRNA	10*7.2*3.6	12,377
25	10w	Female	s.c	Bcl9-shRNA	9*7.4*3.7	16,898
29	10w	Female	s.c	Bcl9-shRNA	8.4*7.6*3.8	13,230

### Specimen Processing

Fresh tumors were collected in MACS Tissues storage solution (130-100-008, Miltenyi Biotec) in the operation room after surgical resection and immediately transferred to the laboratory for processing. Tissues were minced into <1mm^3^ on ice, transferred to a C tube (130-093-237, Miltenyi Biotec) and enzymatically digested with MACS Tumor Dissociation Kit (130-095-929, Mitenyi Biotec) according to corresponding programs. The resulting suspension was filtered through a 40 μm cell strainer (Falcon) and washed by RPMI 1640(C11875500BT, Gibco). Erythrocytes were removed by adding 2 ml Red Cell Lysis Buffer (555899, BD bioscieces). A Dead Cell Removal Kit (130-090-101, Miltenyi Biotec) was subsequently used to enrich live cells. After re-suspended in RPMI 1640 (C11875500BT, Gibco), single-cell suspension was obtained. Trypan blue (15250061, Gibco) was next used to check whether cell viability was >90% to be qualified enough for library construction.

### 10X Library Preparation and Sequencing

Cell concentration was adjusted to 700–1,200 cells/μl to run on a Chromium Single-Cell Platform (10x Genomics ChromiumTM). 10x library was generated according to the manufacturer’s protocol of 10X genomics Single Cell 3’ Reagent Kits v2. The clustering was performed on a cBot Cluster Generation System with TruSeq PE Cluster Kit v3. Qubit was used for library quantification. The final library was sequenced on an Illumina HiSeq3000 instrument using 150-base-pair paired-end reads.

Single cell analysis: Raw data were normalized by using CellRanger (version 4.0). The percentage of reads with the correct barcode is above 85%, and the percentage of bases with a quality score greater than or equal to 30 in the barcode sequence is above 95%; the number of high-quality cells were lisetd in **Table 1**. The total number of Unique Molecular Identifiers (UMIs) per cell was calculated for the number of UMI sequences in the sample with standardizing the data and for identification of highly variable features. The median UMI is 7,000. Then, data was rescaled according to cell cycle related genes and remove batch effects by using Seurat function FindIntegrationAnchors and IntegrateData (36). Then use principal component analysis (PCA) to reduce the dimension. Next, Find the neighbors of each cell by embedded K-nearest neighbor (KNN) graph, and then use the Louvain algorithm to cluster the cells, and then project the results of the clustering on the dimension reduction results from embedded tSNE (t-Distributed Stochastic Neighbor Embedding) and Umap (Uniform Manifold Approximation and Projection) (36, 37). Mark each cell population with known markers, and then extract the endothelial cells and fibroblasts subpopulations (Cluster 7 and 8) for further Re-clustering analysis.

Pseudotime analysis was done using Monocle2 package Built-in Reversed graph embedding method (38–40). Differential gene analysis along Pseudotime was done by using the Branched expression analysis modeling (BEAM) function.

### BCL9-Endo-Score

Use formula (1) to calculate the BCL9-endo-Score of each cell or each patient.

(1) $$\text{BCL}9-endo-Score=\frac{GSVA\;\left(BCL9\;\text{Turge gene list}\right)}{GSVA\;\left(\text{BCL}9\;\text{False gene list}\right)+\;0.0000000001}$$

In the formula (1), *GSVA*() is the function of Gene Set Variation Analysis; BCL9 Ture gene list and BCL9 False gene list respectively represent genes enriched in tumor endothelial cells that have been interfered with or not interfered with BCL9. BCL9 Ture gene list: IGFBP7, SPARC, RARRES2, BGN, LOXL1, COL5A2, FSTL1, COL6A2, DCN, MFAP5, SERPING1, AEBP1, GPX3, THY1, MMP2, BMP1, FBN1, ADAMTS2, COL1A1, COL6A3, RCN3, FBLN2, PLPP3, LOXL2, CD248, COL6A1, PDSSTN, RNASE4, COL3A1, COL1A2, COL5A3, C1QTNF6, MGST1, SERPINF1, SOD3, EBF1, EFEMP2, CYGB, SULF1, FXYD1, VCAN, NBL1, FN1, TGFBR2, SERPINA3, SELENOM, MMP14, RCN1, GPX7, BICC1; BCL9 False gene list: TCP1, RPL12, RPL3, RPL4, TMPO, RPL7, PRKG2, RRM2, LARS2, FCER1G, RAD21, EZR, MTAP, CD9, RPS6, TOP2A, HSP90AB1, HSPA9, MT-CYB, HBEGF, AMIGO2, ACTN4, ACTB, CAVIN2, PLA2G7, CENPF, ATP5F1B, HSPA8, EEF2, TUBA1C, RPS18, ANLN, RAN, WDR31, NOLC1, CPE, TM4SF1, HSPD1, SPP1, PHGDH, TUBA1B, S100A4, CD74, UBE2C, LGALS7, HMGB2, CAV2, ESM1, CCND1, HMGA1.

### Gene Prognostic Performance in The Cancer Genome Atlas Samples

The TCGA datasets were downloaded from Xena Functional Genomics Explorer (https://xenabrowser.net/). The samples were divided into high and low expression groups based on Maxstat (maximally selected rank statistics) algorithm. A Kaplan-Meier curve was constructed to compare the overall and disease-free survival in two groups. Log-Rank P value and HR were also calculated.

### Gene Set Variation Analysis, Gene Set Enrichment Analysis, and Metascape Analysis

GSVA analysis adopts the corresponding gene list of different sample combinations from single cell data or TCGA data to complete with R software.

GSEA analysis uses the grouping information obtained by single-cell cluster analysis, and uses the C5 signal pathway in the Molecular Signatures Database v7.1 to annotate (41, 42).

The Metascape analysis is done with reference to the reported method (43).

## Results

### Clustering Analysis of 12 Samples

**Figure 1:**
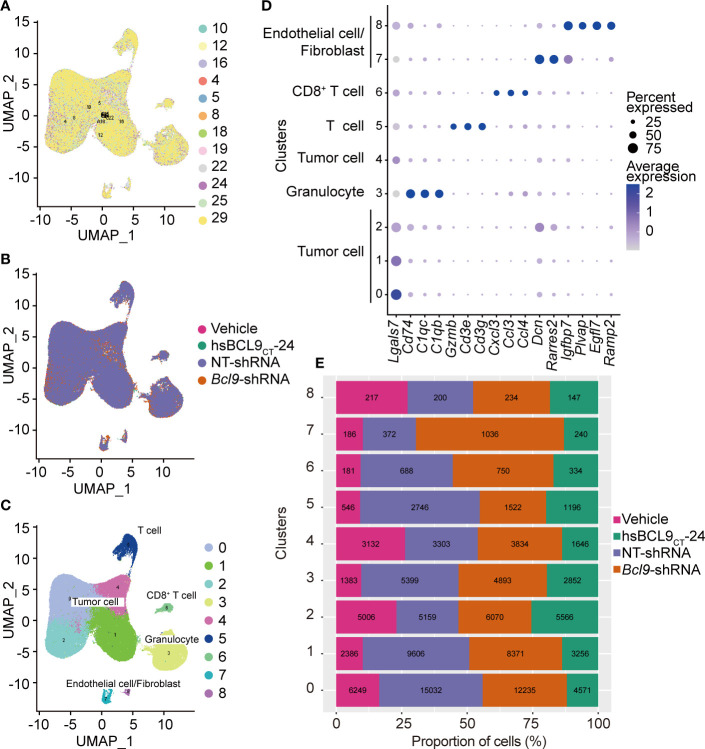
UMAP and cell cluster analysis. **(A)** UMAP of the samples profiled, and UMAP of the associated cell type. Color coded by their associated samples. **(B)** UMAP analysis of sample types of origin (vehicle, hsBCL9CT-24, NT, BCL9-shRNA). **(C)** UMAP of all cells profiled, and UMAP of the associated cell type and clusters. Color coded by their associated clusters. **(D)** Bubble chart of the eight clusters. **(E)** The fraction bar plot of eight clusters originating from vehicle, hsBCL9CT-24, NT, BCL9-shRNA (Numbers show real cell numbers).

In order to study the effect of BCL9 dysfunction on tumor single cell at mRNA levels, we grouped and integrated the BCL9 Genetic deprivation and inhibitor-treated single-cell mRNA sequencing data to remove the batch effect. The further standardized data are clustered using UMAP algorithm and adjacent algorithm, displayed in two dimensions of UMAP graph, and marked with sample identifications or processing groups, as shown in **Figure 1A**. The results show that most of the cells from different samples were evenly distributed among different clusters, which indicated that the effect of batch effect processing and the data quality had reached the level at which they could be combined and analyzed. The results of the cluster analysis of the adjacency algorithm are further displayed on the UMAP graph, **Figure 1C**. We performed the data comparison in the cell annotation database and the differential expression analysis between different clusters in **Figure 1C** to label the cell types. Results showed that all of the samples could be classified as tumor cells, T cells, CD8 T cells, granulocytes, and endothelial/fibroblast cell types are shown in **Figures 1D, E** and **S1**.

To study the perturbation effects of different treatments (BCL9 KD, RNAi control; BCL9 inhibitor or control) on different clusters of cells, we marked the processing group information on the UMAP diagram (**Figure 1B** and **Figure S2A**, **B**). The results showed that most cell clusters did not differ significantly across different treatment groups, but in 7–8 clusters, that is, endothelial/fibroblast clusters, different groups showed different distributions. The four groups are not well merged together, but dispersed (**Figure S2B**), indicating that the two clusters of cells have high heterogeneity. which suggests these two clusters of cells are affected by different treatments.

### Re-Clustering and Transcription Analysis of Endothelial Cell

**Figure 2:**
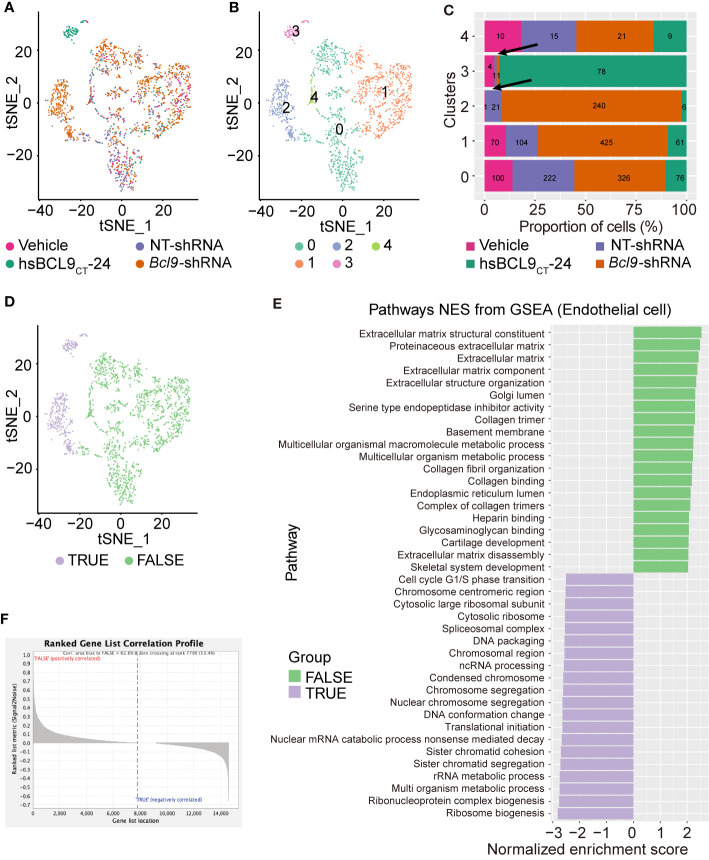
UMAP and GESA analysis of endothelial cell. **(A)** UMAP plot, Sample type of origin (vehicle, hsBCL9CT-24, NT, BCL9-shRNA). **(B)** Four different clusters by UMAP analysis. **(C)** The fraction bar plot of four clusters originating from vehicle, hsBCL9CT-24, NT, BCL9-shRNA (Numbers show real cell numbers). **(D)** UMAP plot of FLASE and TRUE groups. **(E)** GSEA analysis on FLASE and TRUE groups. **(F)** Rank gene correlationship profile of FLASE and TRUE groups.

In order to further determine whether the endothelial/fibroblast clusters show different treatment-dependent clustering effects, we performed re-clustering analysis on cell clusters 7–8 using the tSNE algorithm. The results are shown in **Figures 2A–C** and **S2**. The re-clustered clusters 2 and 3 of endothelial/fibroblasts are visibly distinct from those of the main population, **Figures 2A–C**. The cells treated with BCL9 KD and BCL9 inhibitors were significantly enriched in clusters 2 and 3. This indicates that treatment with BCL9 KD and BCL9 inhibitors has significantly changed the expression profiles of 2 and 3 clusters of endothelial/fibroblasts.

In order to further study the specific biological processes by which BCL9 KD and BCL9 inhibitor treatment change the expression profiles of clusters 2 and 3 of endothelial/fibroblasts, we performed gene set enrichment analysis (GSEA) on cells belonging to these two clusters of endothelial/fibroblast clusters (True) and those not belonging to these clusters (False). As shown in **Figures 2D, E**, the results show that they do not belong to clusters 2 and 3. The list of genes related to such endothelial cell functions as the establishment of extracellular mechanisms was enriched in the cells, while the cells belonging to clusters 2 and 3 did not show this enrichment effect. Rather, they showed some background differences such as ribosomal changes and metabolic changes. These results indicate that treatment with BCL9 KD and BCL9 inhibitors changes the function or differentiation status of endothelial/fibroblasts. From the results of GSEA, we selected the top 50 genes with high enrichment scores in the True and False clusters and named them the BCL9 True gene list and the BCL9 False gene list, as shown in **Figure 2F**.

### Pseudo-Time Analysis of Endothelial Cells

**Figure 3:**
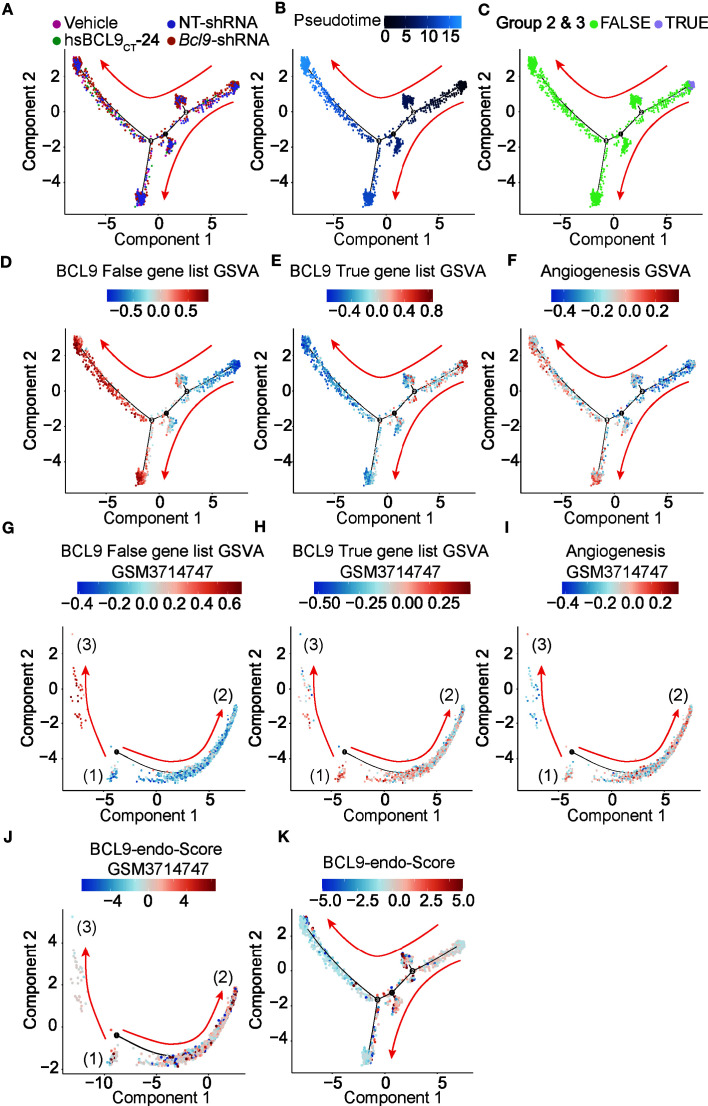
Pseudotime analysis of endothelial cell. **(A–F, K)** Development trajectory of other types of endothelial cells and fibroblast populations along pseudo-time in two-dimensional space in vehicle, hsBCL9CT-24, NT, BCL9-shRNA. **(G–J)** Development trajectory of other types of endothelial cell population from published dataset (GSM3714747). Each point corresponds to a single cell and is color-coded by cell subgroup and differentiation score. Arrows indicate the direction of differentiation.

We performed a simulation analysis of endothelial cells/fibroblasts to further study the effect of BCL9 KD and BCL9 inhibitor treatment on the function and differentiation of endothelial cells/fibroblasts. As shown in **Figures 3A–C**, endothelial cells/fibroblasts can be divided into three branches; those belonging to two clusters 2 and 3 endothelial cells/fibroblast clusters (True) are mainly distributed on the right and upstream of the second node, as shown in **Figure 3C**. We show the gene set variation analysis (GSVA) rankings of the BCL9 True gene list and the BCL9 False gene list on the graph of the pseudotime analysis. As shown in **Figures 3D–F**, the cells gradually differentiated from node 1 along two opposite paths. At nodes 2 and 3, the GSVA score of the BCL9 True gene list was higher near node 2, and the GSVA score of the BCL9 False was higher near node 3. The GSVA score of the angiogenic gene list near 3 nodes was also relatively high. This shows that the effect of BCL9 KD and BCL9 inhibitor treatment on endothelial cells may involve angiogenesis. The heat map constructed by the BEAM function shows the changing trend of key genes along pseudotime (**Figure S3** and **S4**).

In order to further study the effect of BCL9 KD and BCL9 inhibitor treatment on endothelial cells at specific stages of endothelial cell differentiation, we reanalyzed the published mouse liver database (GSM3714747) (44). Liver endothelial cells undergo all of the stages of endothelial cell differentiation, including three stages of relatively primitive cell types (1), well-differentiated sinusoidal endothelium (2) and cells similar to astrocytes (fibroblasts) (3). The entire process of endothelial differentiation can be shown. The gene lists BCL9 True and BCL9 False found in the treatment of BCL9 KD and BCL9 inhibitors were used in mouse liver endothelial data for GSVA analysis, and the GSVA scores are shown in terms of the results of the pseudo-time reanalysis (**Figures 3G–I**). The results showed that the BCL9 False gene list was mainly enriched in astrocyte-like cell clusters (3), while the BCL9 True gene list was mainly enriched in the relatively primitive cell clusters (1).On both sides, we also observed weak enrichment of the BCL9 True gene list, but the enrichment trend is obviously biased to the side of cell clusters (2). This shows that the treatment of BCL9 KD and BCL9 inhibitors can block endothelial cells transition from cell clusters (1) to both ends. The angiogenesis genes are enriched in well-differentiated sinusoidal endothelium cell clusters (2) and cell clusters (3). Based on these results, we can conclude that treatment with BCL9 KD and BCL9 inhibitors can differentiate endothelial cells into the branch of astrocyte-like fibroblasts or highly differentiated endothelial cells.

### BCL9 Endothelial Function Score (BCL9-Endo-Score) and Clinical Significance

In order to further study the clinical significance of BCL9 KD and BCL9 inhibitor treatment on endothelial/fibroblasts, we propose a BCL9 endothelial function Score (BCL9-endo-Score). BCL9-endo-Score is defined as GSVA (BCL9 True gene list)/GSVA (BCL9 False gene list + 0.0000000001), plotted in **Figures 3J, K**. We can see that the cells with high BCL9-endo-Score are some types of cells between the more primitive cells and differentiated cells, which are more inclined to the angiogenic side. This reflects the essential connotation of BCL9-endo-Score, which is the potential of cells to focus on angiogenesis but not on the secretion of extracellular matrix.

**Figure 4:**
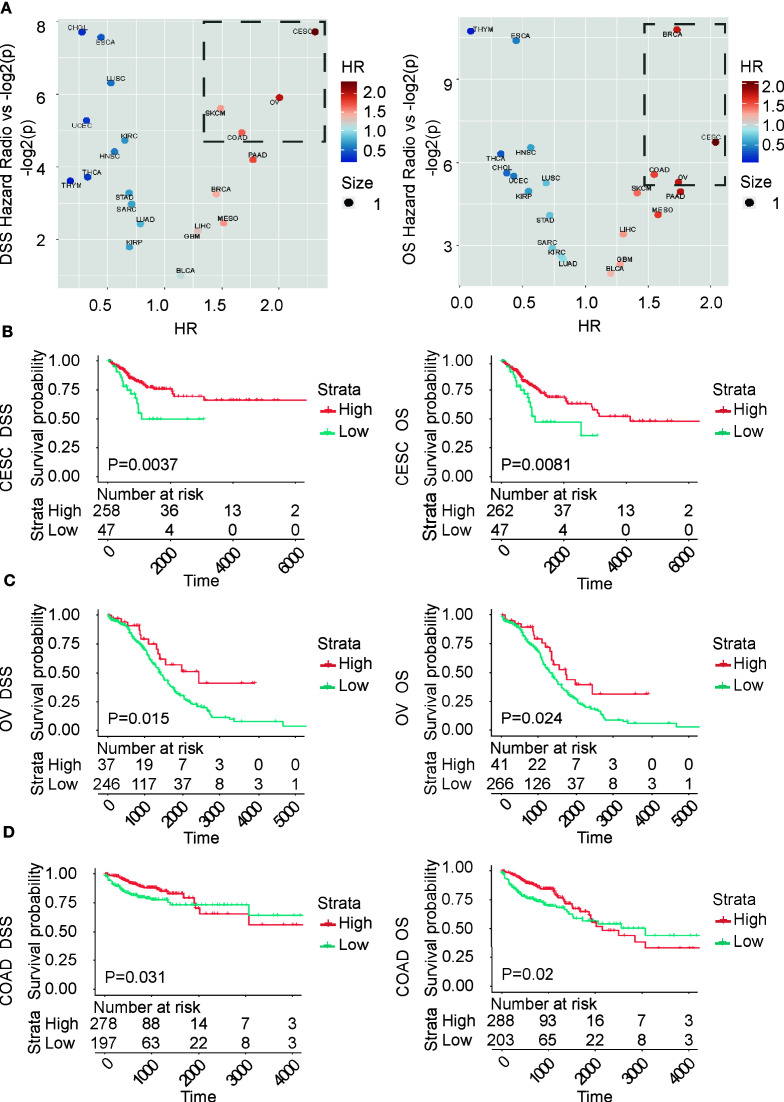
BCL9-endo-Score can predict the prognosis of TCGA tumor patients according to the traditional classification. **(A)** Volcano chart of hazard ratio (HR) of different types of cancer. **(B–D)** Survival analysis chart of TCGA patients according to traditional classification. OS and DSS respectively represent overall survival and disease-specific survival rate.

In this project, we also will use the BCL9-endo-Score to score all of the cancer samples in the TCGA database. We performed survival analysis for each cancer type according to the BCL9-endo-Score. These results show that, in most cancer types, patients with high BCL9-endo-Scores have significantly poor prognosis, **Figures 4A–D**. This shows that the effects of BCL9 KD and BCL9 inhibitor treatment on the function and differentiation of endothelial cells/fibroblasts can affect the prognosis of tumor patients, and this may also lay an experimental foundation for clinical medication.

**Figure 5:**
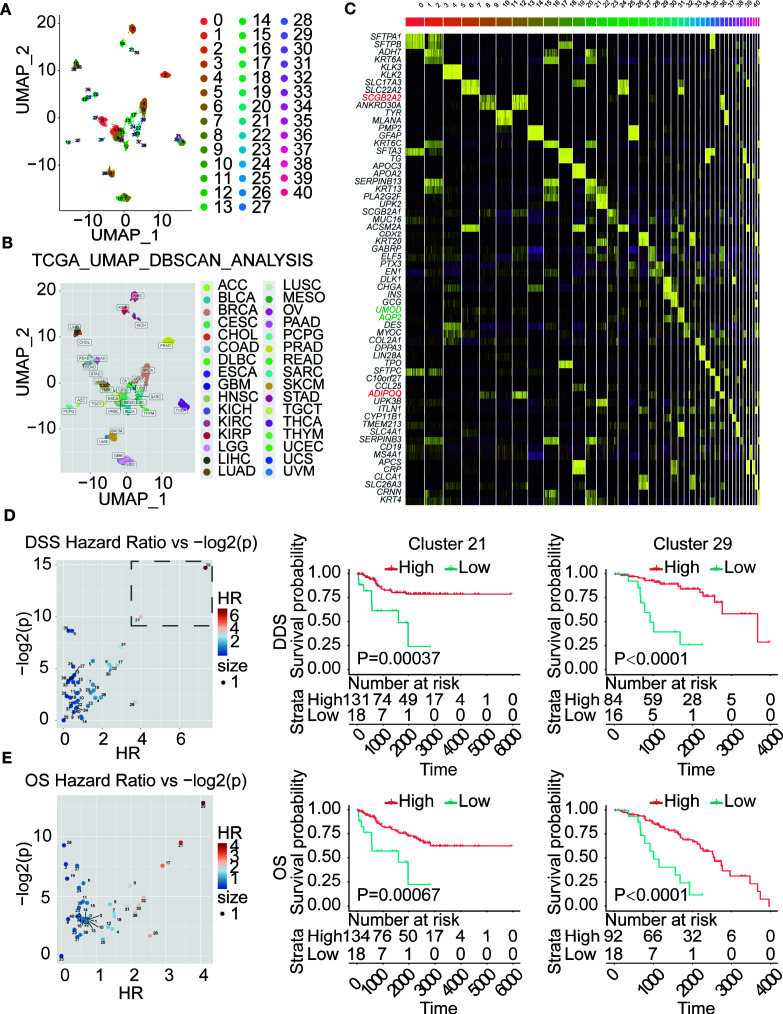
BCL9-endo-Score can predict the prognosis of TCGA tumor patients clustered by gene expression. **(A)** Cluster analysis of TCGA patients according to gene expression patterns. **(B)**. to label with traditional classification **(C)**. Differential gene analysis of patients in different clusters. **(D, E)**. Survival analysis of TCGA patients in different clusters. On the left is the Volcano chart of hazard ratio (HR) of different patient clusters. The survival analysis is on the right.

In order to further study the clinical significance of BCL9-endo-Score, we performed Louvain algorithm cluster analysis and UMAP reduction of dimensions for all TCGA tumor patients based on their mRNA expression similarity. The results of the cluster analysis are displayed on the UMAP graph, **Figures 5A–C**. It can be seen that the entire TCGA patients are divided into 40 clusters. These clusters are basically attributable to the site of cancer, but there are also some tumors from different sites that are clustered together, or some subgroups are produced. In order to further clarify the marker gene of each cluster. We have performed differential gene analysis on TCGA cluster, and the results are shown in the figure.

Then we use each cluster as a group, use BCL9-endo-Score to score each patient, and then use BCL9-endo-Score to group the patients in the cluster and use the Maxstat (maximally selected rank statistics) algorithm for survival analysis, **Figures 5D, E**. The results showed that both cluster 21 and 29 showed a strong and significantly high-risk ratio HR. Among them, cluster 29 is composed of part of BRCA (103) and NHSC (7), and cluster 21 is composed of part of KIRC (90), KIRP (37), KICH (25), and SARC (1). Through the differential genes, we can see that the markers of cluster 29 and cluster 21 are SCGB2A2, ADIPOQ gene and UMOD and AQP2 gene, respectively, **Figure 5C**. The above results indicate that in the above-mentioned tumors BCL9-endo-Score can well judge the clinical prognosis.

## Discussion

### Classification of Tumor Endothelial Cells

Endothelial cells are a type of cells derived from the mesoderm. On the histological level, endothelial cells form the inner walls of various capillaries and large blood vessels in the tissue (45). There is no doubt that endothelial cells play an important role in physiology and pathology. Many important diseases are related to disorders of the structure and function of endothelial cells, such as heart disease, diabetes, high blood pressure, liver cirrhosis, pulmonary fibrosis, and so on (45). In tumors, endothelial cells are generally considered to be involved in the formation of blood vessels and are responsible for the communication of material information inside the tumor and outside the tumor (46). Endothelial cells generally develop from mesenchymal stem cells and have a general marker of fibroblasts and mesenchymal stem cells. We grouped single cells of mouse colon cancer cells treated with BCL9 KD and BCL9 inhibitors according to the similarity of their mRNA expression, and found that the two groups 7 and 8 have markers related to endothelial cells and fibroblasts. Among them, many markers are shared by endothelial cells and fibroblasts. For example, the highly expressed marker Ccl4 in the 7 population has been reported to play an important role in the differentiation of endothelial cells, and collagen Col1a1 is considered to be the marker of endothelial cells (47). Gpihbp1 (48), Egfl7 (49), and Plvap (50) in the eight groups are also considered to be important markers of endothelial cells. Among them, Plvap is considered to form the membrane of glomerular endothelial cells, and belongs to the marker of endothelial differentiation (50). At the same time, Col3a1 (51) in group 7 and Pi16 (52) in group 8 were reported to be fibroblast markers. Therefore, in summary, endothelial cells and fibroblasts share the characteristics of a common marker. In other words, it is difficult to distinguish and define which cells are endothelial cells and which cells are fibroblasts. This result is consistent with previous reports. Fibroblasts and endothelial cells are not only the same in source, but may have the properties of mutual differentiation and transformation. In the UMAP cluster analysis graph of our result, we can observe that the 7 and 8 groups have a connected topological relationship, which also proves the above point.

### Functional Differences Between Different Tumor Endothelial Cells

The function of endothelial cells in tumors has been explored for a long time. Even inhibiting the growth of tumor endothelial cells is a very potential target in tumor therapy. On the one hand, the massive growth of endothelial cells promotes the blood supply inside the tumor (27), so that the internal nutrition of the tumor is sufficient, the tumor grows faster, and the tumor mass is larger, oppressing the surrounding organs and crowding out normal cells; on the other hand, a large number of endothelial cells make the internal oxygen supply of the tumor sufficient, avoiding some EMT transition or metastasis caused by hypoxia (53). Therefore, endothelial cells have dual functions in tumors, and may have completely opposite functions in tumors of different nature. Our research results found that the loss of BCL9 function can make the tumor endothelial cell and fibroblast population clearly grouped. In this type of tumor endothelial cells and fibroblasts derived from BCL9 functional loss samples, the biological processes related to the formation of extracellular matrix have undergone significant changes. In the endothelial cell population with relatively normal BCL9 function, the significantly enriched genes are concentrated in the establishment of extracellular matrix, Golgi lumen function, collagen fibrous tissue, ER lumen function, connective tissue development and bone system development. Blood vessels have always been regarded as an important type of connective tissue in histology. The construction of the special tubular structure of blood vessels is highly dependent on the construction of extracellular matrix (54); the blood vessel walls are arranged with highly organized collagen fibers (27) to maintain the elasticity of blood vessels. At the same time, the extracellular matrix constitutes the basement membrane of the capillaries (54). As we all know, extracellular matrix proteins are a type of typical secreted proteins, which are processed by the endoplasmic reticulum and Golgi apparatus, and finally reach the caveolae and other vesicle systems and are finally secreted outside the cell (55). In summary, the biological function of BCL9 relatively normal endothelial cell population is highly overlapped with the formation of extracellular matrix. However, in the endothelial cells and fibroblast populations whose BCL9 function is affected, that is to say, the endothelial cells and fibroblasts enriched in the samples treated with BCL9 KD and BCL9 inhibitors are not enriched with the formation of extracellular matrix. Instead, it is the enrichment of some related biological processes such as ribosomes, metabolism and cell chromosome formation. Instead, some ribosomes, metabolism, and cell chromosome formation are closely related to the cell cycle. Although the entire analysis process has been corrected for mitochondrial genes and cell cycle, these differences still exist in BCL9 KD and BCL9 cells and their corresponding control cells, which may imply that the mechanism of BCL9’s effect on endothelial cell function may be related to the cell cycle. Our further enrichment analysis of these genes with Metascape also shows that cell cycle-related signaling pathways R-HSA-69278 from Reactome are among the best (43, 56), **Figure S5**.

### Normal Endothelial Cells Two Kinds, Tumor Endothelial Cells One Kind

**Figure 6:**
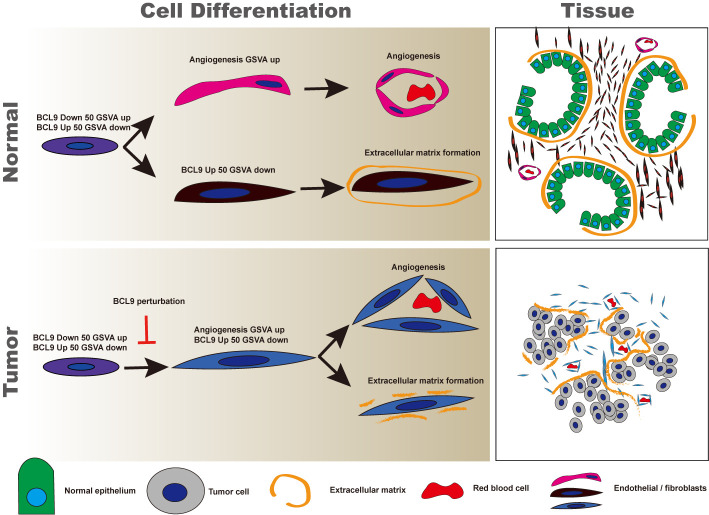
Diagram of the development pattern of endothelium in normal and tumor tissues. It can be seen that fibroblasts and endothelial cells of normal endothelial cells differentiate along two different paths, and ultimately perform different functions. Supplementary File 1 Data of TCGA patients grouped by BCL9-endo-Score (traditional classification).

Summarizing the mathematical significance of our proposed BCL9-endo-Score (BCL9-endo-Score), it can be explained as the biggest difference in gene expression between loss-of-function endothelial cells and normal endothelial cells, but this difference does not only consider high expression and low expression (**Figure 6**). And we further use the GSVA algorithm to introduce the generation of gene expression changes to cover the possibility of the opposite gene expression situation due to the complementation of functions in the occurrence of real signal pathways. In the virtual time analysis of the endothelial cell population, it can be seen that BCL9-endo-Score and the traditional signal pathway “angiogenesis” have a certain overlap in the development of tumor endothelial cells. However, in the normal endothelial cell population, BCL9-endo-Score is present in the endothelial cell population with an intermediate degree of differentiation, which is similar to the cell population between astrocytes and the most differentiated sinusoidal endothelial cells in the liver. With high scores, these cells also show high “angiogenesis” ability. In other words, most of the endothelial cells that function normally in tumors are endothelial cells similar to astrocytes in the liver. These endothelial cells may not have the ability to form normal blood vessels. This means that the cells with a higher BCL9-endo-Score may be the most primitive cells in the entire endothelial cell population. In normal tissues, they differentiate into cells capable of “angiogenesis” and incapable of vascularization. In tumors, cells differentiated from BCL9-endo-Score high-scoring cells all have the ability to angiogenesis. In other words, the enrichment of BCL9-endo-Score cells in the tumor may mean that the tumor’s angiogenesis is hindered.

### Relationship Between Clinical Output and Pathological Characteristics

In the TCGA database, we found that the high BCL9-endo-Score indicates a good prognosis in three cancers: COAD, CESC and OV. Angiogenesis inhibitory therapies have been extensively reported and reviewed in the treatment of COAD, CESC and OV, suggesting that tumorigenesis and development depend on angiogenesis. But because the HR we calculated is about 2, it is a medium level. The reason may be that the classification method that depends on the tumor site may not reflect the nature of the tumor cells. Therefore, this classification method based on the tumor site may be less helpful in predicting the prognosis of the tumor.

In the results of our cluster analysis based on TCGA, and then survival analysis based on different categories. We observed HRs with OS exceeding 3 and HRs with DSS exceeding 4, showing a high-risk ratio. In terms of tissue type, the 21 groups with the second highest HR, most of which are composed of three types of kidney cancer. Renal cell carcinoma (KICH, KIRC, KIRP), due to the large distribution of blood vessels in renal cell carcinoma, the important role of angiogenesis in renal cell carcinoma is not difficult to understand. However, the 29 groups with the highest HR consist of some of the BRCA and NHSC that are positive for the SCGB2A2, ADIPOQ gene. Among them, the SCGB2A2 gene is a milk-specific immunoglobulin, and ADIPOQ is the gene of adiponectin. Adiponectin in adults is negatively correlated with fat reserves. In other words, it may be correlated with thin body shape. Because BRCA belongs to the cancer of the glands in the connective tissue inside the skin, and the breast may require a large amount of material exchange when synthesizing and secreting milk, so that it is given to breast the potential of cells to regulate angiogenesis. While NHSC occurs in the skin, it is likely that a small part of it has some similarities with breast development, so it is classified into one category. These results need to be verified by histopathological methods in subsequent studies.

## Data Availability Statement

The datasets for this study can be found in the NCBI (Accession: PRJNA675146) and Figshare (https://figshare.com/s/edbc79f329291b06e976). The code involved is published on Github (https://github.com/weizhuang128/bcl9_endo).

## Author Contributions

ZWe analyzed the data, prepared the figures, and wrote the manuscript. WC prepared the figures and edited the manuscript. MF and SS prepared the samples. DZ conceptualized and wrote the manuscript. All authors contributed to the article and approved the submitted version.

## Funding

The current study was supported by the Science and Technology Commission of Shanghai (18ZR1403900) (DZ), the National Natural Science Foundation of China (81872895) (DZ), and the project on joint translational research in Shanghai Institute of Materia Medica and Fudan University (FU-SIMM20181010) (DZ). Fudan School of Pharmacy and Pudong hospital Joint Research Fund (RHJJ2018-03 to TY and DZ), Shanghai Science and Technology Commission (18JC1413800 to BZ and DZ).

## Conflict of Interest

The authors declare that the research was conducted in the absence of any commercial or financial relationships that could be construed as a potential conflict of interest.

## Supplementary Material

Supplementary Figure 1 Heatmap of the eight clusters. Columns, individual cells; rows, genes (Top20).

Supplementary Figure 2 The analysis process of re-clustering. **(A)** The UMAP plot of all cells shows the expression of marker gene in the endothelial cells and fibroblast populations; **(B)** the enlarged display of the local populations, the arrow shows the areas that are not merged; the re-clustering analysis of the endothelial cells and fibroblast populations, **(C) (D)** show UMAP and tSNE plots, sample type of origin (vehicle, hsBCL9CT-24, NT, BCL9-shRNA), **(E) (F)** shows the UMAP and tSNE plots of re-clustering analysis results of six different clusters. The dotted lines select clusters with similar topological relationships.

Supplementary Figure 3 The trend of the gene expression generated by the BEAM function along pseudotime of endothelial cells and fibroblast populations.

Supplementary Figure 4 The trend of the gene expression generated by the BEAM function along pseudotime of endothelial cell population from published dataset (GSM3714747).

Supplementary Figure 5 Signal pathway enrichment analysis based on Metascape.
