# Supplementary material for: Bcl9 Depletion Modulates Endothelial Cell in Tumor Immune Microenvironment in Colorectal Cancer Tumor
Source: Front Oncol. 2021 Jan 19;10:603702. doi: 10.3389/fonc.2020.603702 (PMC7856347; doi:10.3389/fonc.2020.603702)
Supplement: Supplementary Figure 1 — Heatmap of the eight clusters. Columns, individual cells; rows, genes (Top20). [file DataSheet_1.pdf]

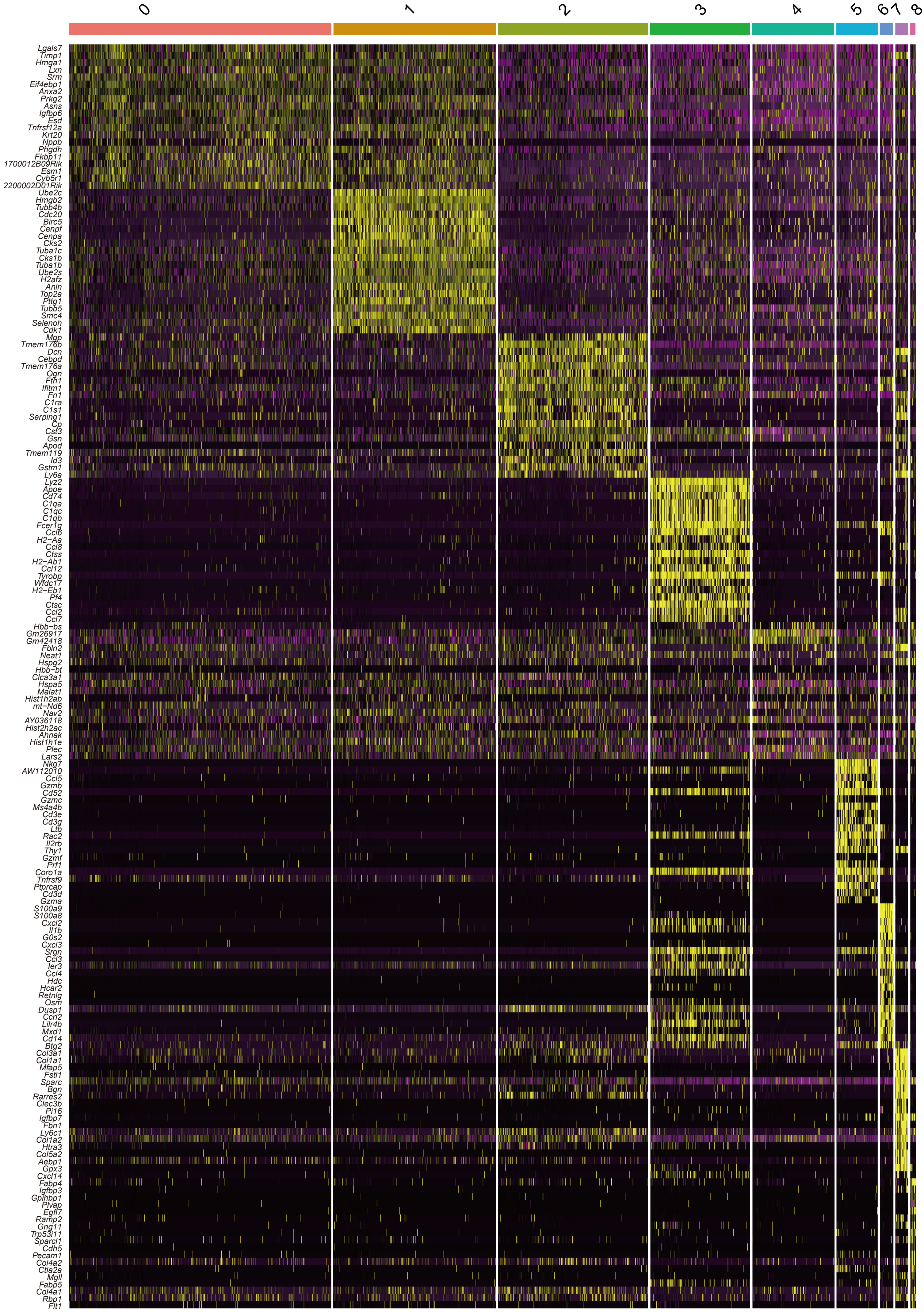

Supplementary Figure 1

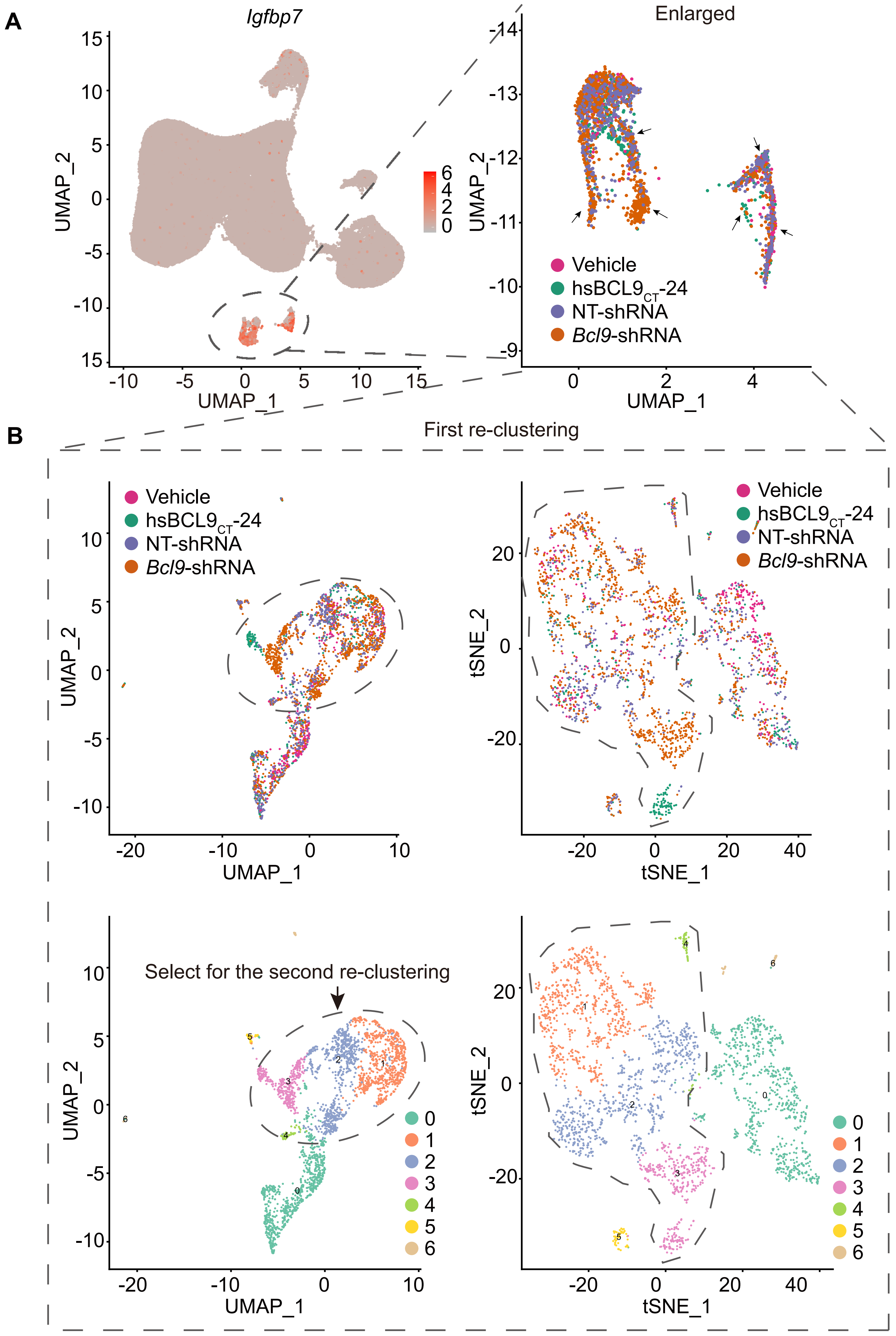

Supplementary Figure 2

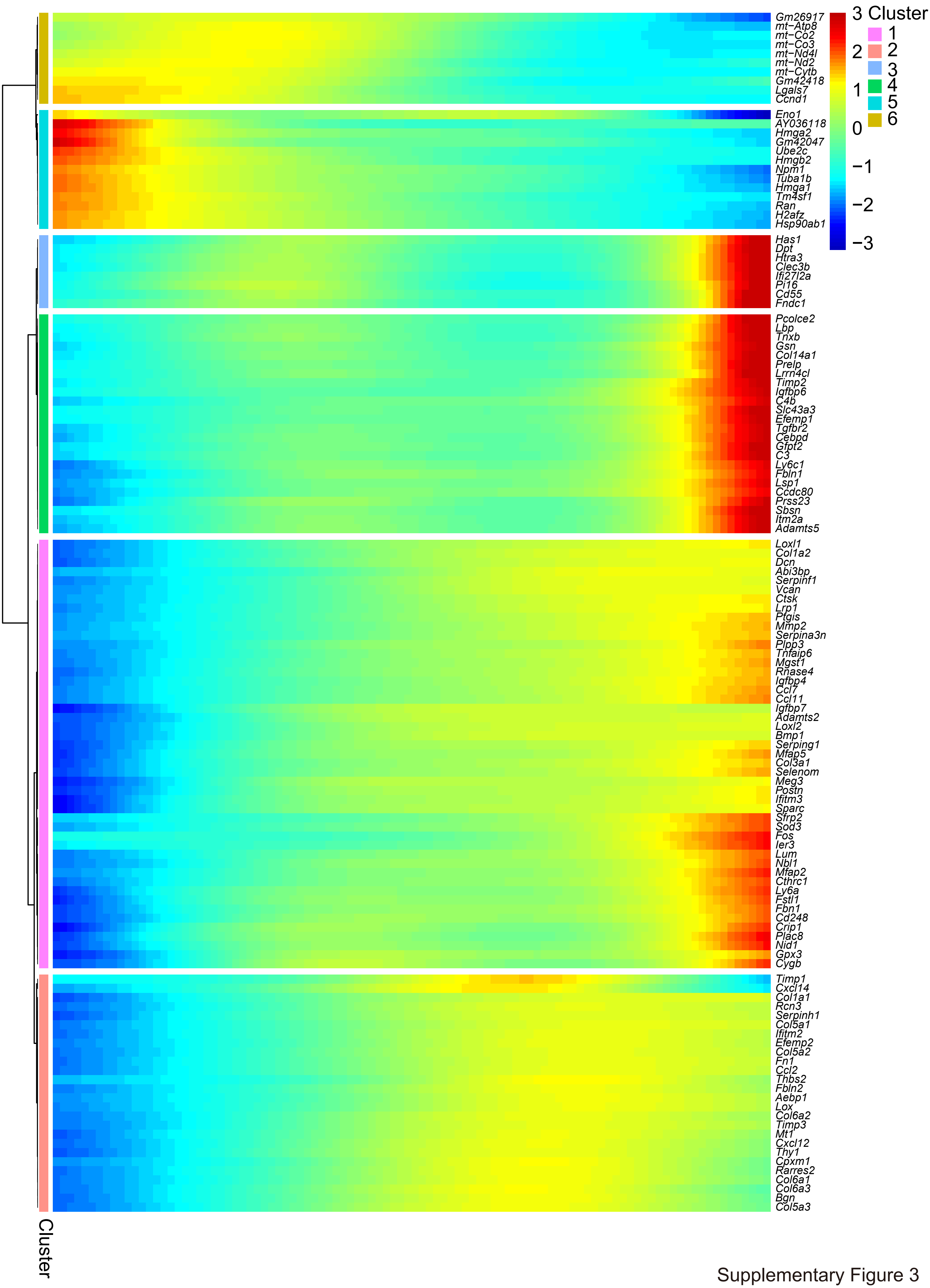

Supplementary Figure 3

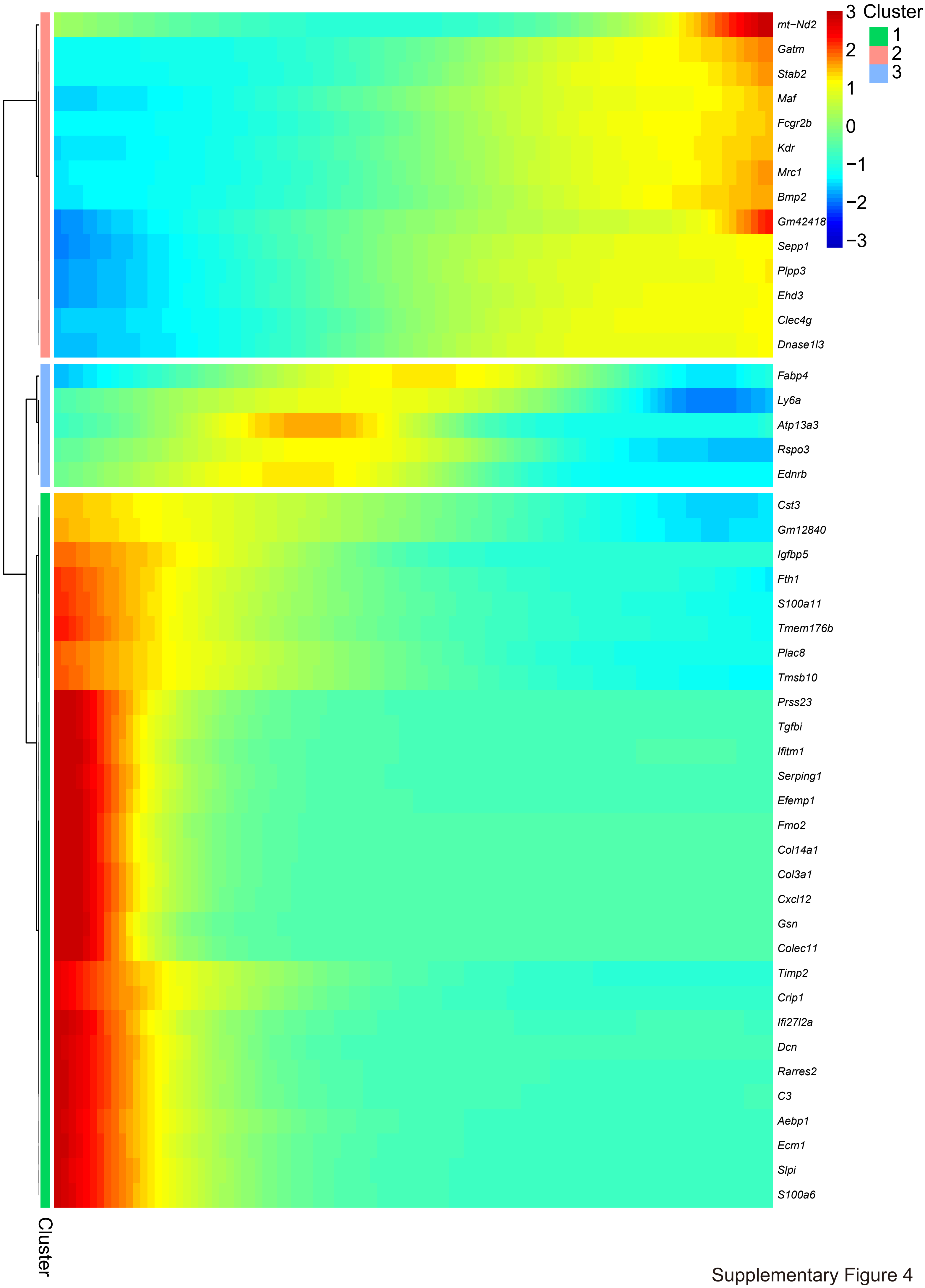

Supplementary Figure 4

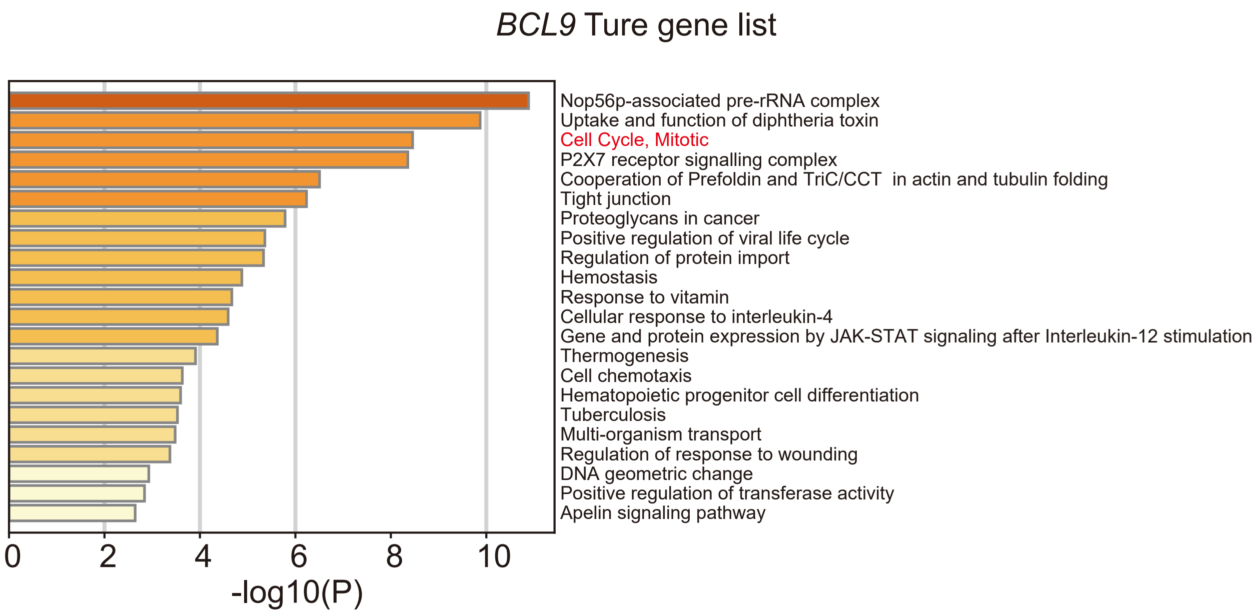

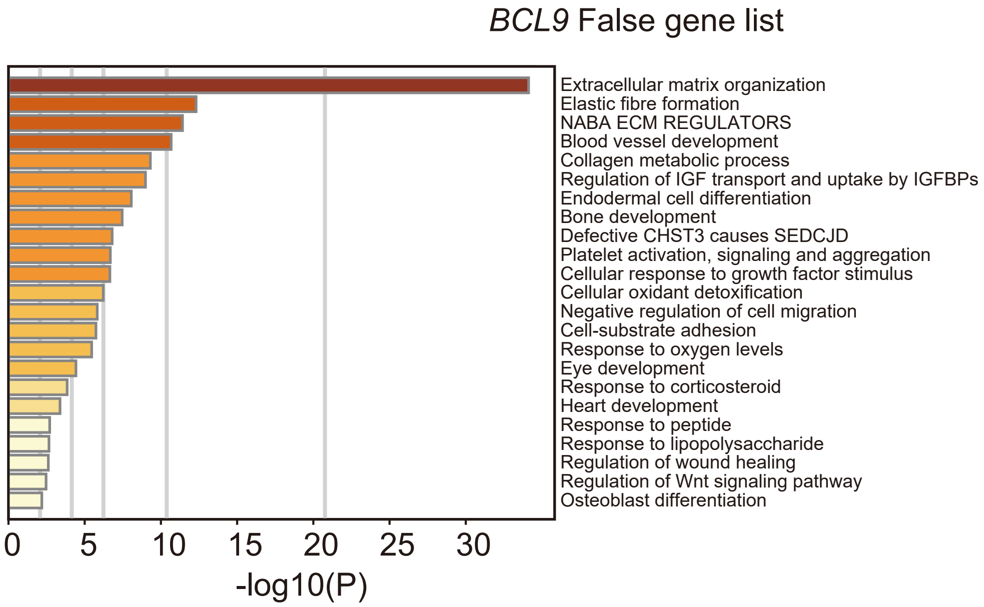

Supplementary Figure 5
